# Trends in the Stage Distribution of Colorectal Cancer During the COVID-19 Pandemic in Japan: A Nationwide Hospital-claims Data Analysis

**DOI:** 10.2188/jea.JE20220347

**Published:** 2024-07-05

**Authors:** Masato Ota, Kohei Taniguchi, Mitsuhiro Asakuma, Sang-Woong Lee, Yuri Ito

**Affiliations:** 1 Department of General and Gastroenterological Surgery, Osaka Medical and Pharmaceutical University, Osaka, Japan; 2 Department of Medical Statistics, Research & Development Center, Osaka Medical and Pharmaceutical University, Osaka, Japan; 3 Translational Research Program, Osaka Medical and Pharmaceutical University, Osaka, Japan

**Keywords:** COVID-19 pandemic, colorectal cancer, cancer stage

## Abstract

**Background:**

The coronavirus disease 2019 (COVID-19) pandemic has affected cancer care. The aim of this study was to clarify the trend of colorectal cancer (CRC) stage distribution in Japan during the COVID-19 pandemic.

**Methods:**

In this retrospective study, we used an inpatient medical claims database established at approximately 400 acute care hospitals. From the database, we searched patients who were identified as having the main disease (using International Classification of Diseases, 10^th^ revision codes [C18.0–C20]) between January 2018 and December 2020. A multivariate logistic regression analysis was used to determine the impact of the pandemic on CRC stage distribution each month, and the odds ratio (OR) for late-stage cancer was calculated.

**Results:**

We analyzed 99,992 CRC patients. Logistic regression analysis, including the interaction term between increased late-stage CRC effect during the pandemic period and by each individual month, showed that the OR for late-stage CRC was highest in July during the pandemic, at 1.31 (95% confidence interval [CI], 1.13–1.52) and also significantly higher in September at 1.16 (95% CI, 1.00–1.35).

**Conclusion:**

We investigated the trend of CRC stage distribution during the COVID-19 pandemic using a nationwide hospital-claims database in Japan and found that the proportion of early-stage cancers tended to decrease temporarily after the state of emergency declaration due to the COVID-19 pandemic, but the effect was only temporary.

## INTRODUCTION

The first case of coronavirus disease 2019 (COVID-19) was confirmed in Wuhan, China, in December 2019; since then, the infection has spread globally, and the pandemic is still ongoing.^1^^,^^2^ Therefore, many governments implemented lockdowns or declared states of emergency to prevent the spread of severe acute respiratory syndrome coronavirus 2.^3^ The first Japanese case of COVID-19 was confirmed on January 16, 2020. Since then, Japan has seen several waves of expansion and contraction of the infection. On April 7, 2020, the Japanese government declared a state of emergency, which was subsequently lifted on May 25, 2020. Facing this unprecedented global pandemic, surgical societies and gastroenterological endoscopy societies addressed the general principles of surgical or endoscopic treatments in relation to COVID-19 infection.^4^^,^^5^ The use of appropriate triage was recommended, and clinicians were urged to consider the postponement or cancellation of gastrointestinal endoscopies or alternative therapeutic approaches, if possible, for the prevention of nosocomial infections that could result in the breakdown of medical care.

While measures are needed to control the number of new COVID-19 cases,^6^ these measures can negatively affect the diagnosis of other major medical conditions. The COVID-19 pandemic has had a major impact on cancer care due to the diversion of healthcare resources to treat COVID-19 patients and changes in the healthcare-seeking behavior of patients with symptoms of cancer.^7^^,^^8^ Some reports on the management of colorectal cancer (CRC) during the pandemic in the United Kingdom showed a significant impact on the number of tests, diagnoses and treatment initiations.^9^ Although there have been reports of changes in the stage distribution and treatment of gastrointestinal cancers in Japan,^10^^–^^12^ these reports are mainly from a limited number of institutions and areas, and there are few nationwide reports.

In this study, we aimed to clarify the stage distribution of CRC in Japan during the COVID-19 pandemic using nationwide hospital-based claims data.

## METHODS

### Study design and data collection

We conducted the study using administrative claims database, which included information from medical institutions employing Japanese healthcare databases of Diagnostic Procedure Combination (DPC) data provided by Medical Data Vision Co. Ltd. (MDV, Tokyo, Japan). Approximately 20% of all hospitals in Japan operate under this DPC system, and MDV collected data from approximately 400 of these facilities. This dataset represents about 25% of acute care hospitals in Japan, and this number has increased during the period covered by this study. The database includes unique identifiers for the hospital; patient age, sex, height, and body weight at admission; smoking index; diagnosis and comorbidities at admission; clinical cancer stage and tumor, node, metastasis (TNM) classification for malignant tumors (according to the 7^th^ and 8th editions of the Union for International Cancer Control); and procedures (coded with original Japanese codes).

We searched patients who were identified as having the main disease using the International Classification of Diseases, Tenth Revision (ICD-10) codes (C18.0–C20) from the categories: main disease name, disease name that triggered hospitalization and invested most medical resources between January 2018 and December 2020. Colon cancer cases were further categorized into two groups: right-side colon cancer (C18.0–18.4; cecum, appendix vermiformis, ascending colon, hepatic flexure of the colon and transversal colon) and left-side colon cancer (C18.5–C18.7; splenic flexure of the colon, descending colon and sigmoid colon). Rectal cancer (C19–20) was classified into one category, and others (C18.8–C18.9) were classified as unknown.

The period before the COVID-19 pandemic was defined as January 2018 to January 2020, while the period during the COVID-19 pandemic was defined as February 2020 to December 2020. The hospital scale was defined as the number of beds for which we created three categories (≤199, 200–499, and ≥500 beds). TNM classification 8th edition was used for the staging of tumors. The stage information accompanied the inpatient data, and many data entries included multiple admissions for the same patient. For the determination of stage, if there was only a single admission or single stage information, that stage information was used. In the case of multiple admissions and multiple instances of stage information, priority was given if: (1) the admission was for treatment purposes; (2) the content includes surgery or endoscopic treatment, admission at the time of their treatment; (3) chemotherapy is included but not surgery or endoscopic treatment, or admission is for the first chemotherapy only; or (4) surgery or endoscopic treatment or chemotherapy is not included, admission was for the first other treatment, and stage information for that case was used. We further classified stages 0–I as early-stage cancer and II or higher as late-stage cancer. Treatment was categorized by procedure code of treatment, administration of chemotherapy during hospitalization, and purpose of hospitalization (eTable 1).

The requirement for informed consent was waived for this study because of the anonymous nature of the data. Study approval was obtained from the Institutional Review Board at Osaka Medical and Pharmaceutical University (approval number: 2020-072-2).

### Statistical analysis

We compared the characteristics of CRC patients before and during the COVID-19 pandemic. Groups were compared using the χ2 test for categorical variables. The percentages of early and late-stage cancer by month were calculated and compared before and during the pandemic. Multivariate logistic regression analysis adjusted for sex, age, body mass index, smoking index, and hospital scale, with interaction terms was used to determine the impact of the pandemic on the distribution of CRC stages each month, and the odds ratio (OR) and 95% confidence interval (CI) for late-stage cancer were calculated. Predicted proportions of late-stage CRC were also calculated by month before and during the pandemic. All analyses were conducted using STATA version 17 (StataCorp, College Station, TX, USA). A *P*-value <0.05 was considered significant.

## RESULTS

We analyzed 99,992 CRC patients between 2018 and 2020. Characteristics of CRC patients in the two periods are shown in Table 1. During the COVID-19 pandemic, there was a slight increase in the percentages of patients under 60 years old from 15.4% to 16.2%, and in their 70s from 35.0% to 35.9%, and those with a smoking history. In terms of trends in treatment choices, laparoscopic surgery continued to be the most popular technique, while use of endoscopic submucosal dissection increased from 9.4% to 11.2%, and robotic surgery increased from 0.6% to 2.6% during the COVID-19 pandemic.

**Table 1.** Colorectal cancer patient characteristics before and during COVID-19 pandemic

	Total		Before COVID-19		During COVID-19		*P*-value
	*n*	%	*n*	%	*n*	%	
Sex							0.28
Male	57,052	(57.1)	39,267	(56.9)	17,785	(57.3)	
Female	42,940	(42.9)	29,692	(43.1)	13,248	(42.7)	
Age, years							<0.001
≤59	15,693	(15.7)	10,654	(15.4)	5,039	(16.2)	
60–69	24,110	(24.1)	17,055	(24.7)	7,055	(22.7)	
70–79	35,316	(35.3)	24,167	(35.0)	11,149	(35.9)	
≥80	24,873	(24.9)	17,083	(24.8)	7,790	(25.1)	
BMI							0.014
<18.5	12,871	(12.9)	8,933	(13.0)	3,938	(12.7)	
18.5–24.9	62,597	(62.6)	43,233	(62.7)	19,364	(62.4)	
25–29.9	19,251	(19.3)	13,179	(19.1)	6,072	(19.6)	
≥30	3,517	(3.5)	2,365	(3.4)	1,152	(3.7)	
Unknown	1,756	(1.8)	1,249	(1.8)	507	(1.6)	
Smoking index, pack year							<0.001
0	54,560	(54.6)	37,975	(55.1)	16,585	(53.4)	
1–49	28,842	(28.8)	19,604	(28.4)	9,238	(29.8)	
≥50	8,222	(8.2)	5,554	(8.1)	2,668	(8.6)	
Unknown	8,368	(8.4)	5,826	(8.4)	2,542	(8.2)	
COVID-19^a^							<0.001
	94	(0.1)	0	(0.0)	94	(0.3)	
Number of beds^b^							<0.001
≤199	6,231	(6.2)	4,448	(6.5)	1,783	(5.7)	
200–499	52,814	(52.8)	36,437	(52.8)	16,377	(52.8)	
≥500	40,947	(41)	28,074	(40.7)	12,873	(41.5)	
Designated cancer care hospital							0.83
	75,482	(75.5)	52,042	(75.5)	23,440	(75.5)	
Tumor location							0.18
Right	36,760	(36.8)	25,250	(36.6)	11,510	(37.1)	
Left	26,468	(26.5)	18,316	(26.6)	8,152	(26.3)	
Rectum	34,822	(34.8)	24,019	(34.8)	10,803	(34.8)	
Unknown	1,942	(1.9)	1,374	(2.0)	568	(1.8)	
Treatment							<0.001
Open surgery	17,261	(17.3)	12,490	(18.1)	4,771	(15.4)	
Laparoscopic surgery	44,119	(44.1)	30,301	(43.9)	13,818	(44.5)	
Robotic surgery	1,248	(1.2)	448	(0.6)	800	(2.6)	
ESD	9,945	(9.9)	6,456	(9.4)	3,489	(11.2)	
EMR	3,695	(3.7)	2,510	(3.6)	1,185	(3.8)	
Other surgery	3,890	(3.9)	2,517	(3.6)	1,373	(4.4)	
Chemotherapy	4,847	(4.8)	3,396	(4.9)	1,451	(4.7)	
Radiotherapy	281	(0.3)	193	(0.3)	88	(0.3)	
Other treatment	13,752	(13.8)	9,939	(14.4)	3,813	(12.3)	
Examination	954	(1.0)	709	(1.0)	245	(0.8)	
Stage							0.003
Early stage	28,042	(28.6)	19,750	(28.6)	8,605	(27.7)	
Late stage	70,024	(71.4)	49,209	(71.4)	22,428	(72.3)	

BMI, body mass index; COVID-19, coronavirus disease 2019; EMR, endoscopic mucosal resection; ESD, endoscopic submucosal dissection.

^a^COVID-19: Number of patients diagnosed with COVID-19.

^b^Number of beds: Number of beds indicating the size of the hospital.

**Figure 1.**
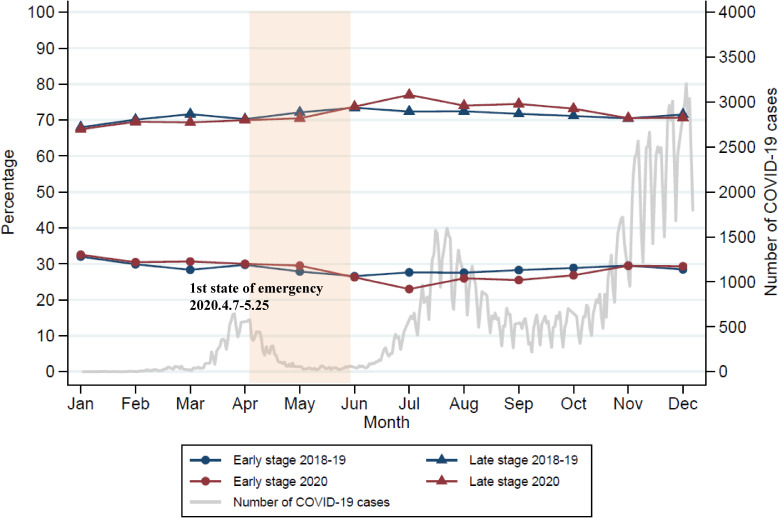
Trend of stage distribution for each month and number of COVID-19 cases in Japan. The period before the COVID-19 pandemic was defined as January 2018 to January 2020, while the period during the COVID-19 pandemic was defined as February 2020 to December 2020. Total colorectal cancer patients were 30,683 in 2018, 33,826 in 2019, and 33,557 in 2020. COVID-19, coronavirus disease 2019.

The trends of percentage of CRC patients by early and late stage per month for 2018, 2019, and 2020, with the number of COVID-19 cases in 2020, are shown in Figure 1 and eTable 2. The percentage of early-stage CRC as a percentage of the monthly total remained around 30%. The first wave of increased COVID-19 infection cases occurred around April, the time of the first state of emergency. There was a second wave of increasing COVID-19 cases from July to August, followed by a sustained decline in the percentage of early-stage CRC.

The results of the logistic regression analysis are shown in Table 2. The OR for late-stage CRC by sex was significantly higher for females than for males, and by month was significantly higher from May to August. Moreover, the analysis, including the interaction term between increased late-stage CRC effect during the pandemic period and by each individual month, showed that the OR for late-stage cancer was highest in July during the pandemic, at 1.31 (95% CI, 1.13–1.52) and also significantly higher in September at 1.16 (95% CI, 1.00–1.35), using February, when the pandemic began, as reference. As a sensitivity analysis, the same analysis was performed for patients whose cancer stage was unknown, when they were classified as early-stage and when they were classified as late-stage. The results of those analyses showed the same trend as the analysis when patients whose cancer stage was unknown were excluded. The late-stage predictions, taking into account the interaction effects, were also high in July, when the largest difference between before and during the COVID-19 pandemic was observed (eTable 3).

**Table 2.** Logistic regression for monthly late stage proportion of colorectal cancer

		Odds Ratio	95% CI	*P*-value
Sex				
Male		Ref		
Female		1.08	(1.05–1.12)	<0.01
Age, years				
≤59		Ref		
60–69		1.13	(1.08–1.18)	<0.01
70–79		1.10	(1.06–1.15)	<0.01
≥80		1.49	(1.42–1.56)	<0.01
COVID-19 pandemic				
Before		Ref		
During		0.99	(0.89–1.09)	0.83
Month				
January		0.90	(0.83–0.97)	0.01
February		Ref		
March		1.07	(0.98–1.17)	0.11
April		1.01	(0.92–1.10)	0.81
May		1.10	(1.01–1.20)	0.03
June		1.17	(1.07–1.27)	<0.01
July		1.10	(1.01–1.20)	0.03
August		1.10	(1.02–1.21)	0.02
September		1.07	(0.99–1.17)	0.11
October		1.04	(0.96–1.14)	0.32
November		1.01	(0.93–1.10)	0.73
December		1.08	(0.98–1.17)	0.10
Interaction term				
(COVID-19 pandemic x Month)				
During	x January	—		
	x February	Ref		
	x March	0.92	(0.80–1.06)	0.24
	x April	1.02	(0.89–1.18)	0.74
	x May	0.93	(0.80–1.08)	0.32
	x June	1.02	(0.88–1.18)	0.78
	x July	1.31	(1.13–1.52)	<0.01
	x August	1.09	(0.95–1.27)	0.23
	x September	1.16	(1.00–1.35)	0.04
	x October	1.12	(0.97–1.29)	0.12
	x November	1.03	(0.89–1.18)	0.72
	x December	0.98	(0.85–1.13)	0.78

CI, confidence interval; COVID-19, coronavirus disease 2019.

Multivariate logistic regression analysis adjusted for sex, age, body mass index, smoking index, and hospital scale, with interaction terms was used to determine the impact of the pandemic on the distribution of CRC stages each month, and the odds ratio and 95% confidence intervals for late-stage cancer were calculated.

## DISCUSSION

This study investigated trends of CRC stage distribution during the COVID-19 pandemic using a nationwide hospital-based claims database in Japan. The impact of the pandemic on the decrease in the proportion of early-stage CRC was found to be particularly large in July and September 2020.

The impact of the COVID-19 pandemic on the OR in late-stage CRC was greatest in July, particularly due to a decrease in early-stage CRC. A previous study in Japan reported that the average monthly number of early-stage CRC cases decreased during the COVID-19 pandemic.^10^ The nature of the database used in this study makes it difficult to evaluate the number of diagnoses alone, but when examined by the distribution of early and late stage percentages, the effects of the state of emergency were delayed by 2 months and occurred at a time when the infection was resurging. Studies in other countries looking at changes in the distribution of CRC stages during the pandemic reported a temporary decrease in early-stage cancers,^13^^,^^14^ and the present study showed similar results.

Colonoscopy is essential for the diagnosis of CRC. The OR for late-stage CRC by month was significantly higher from July to September regardless of the prevalence of COVID-19. The number of CRC screenings is likely to increase in the months following May. The OR for late-stage CRC may be higher during this period because CRC requiring a thorough examination may be detected slightly later, from July to September, and more advanced cancers may be examined more hastily.^15^^–^^17^ Furthermore, the limited testing at medical facilities in the initial stages of the COVID-19 pandemic and the limited colonoscopies required to diagnose CRC may have somewhat delayed the diagnosis of CRC that may have been diagnosed during that time period.^18^ Decreased CRC diagnosis due to restrictions on colonoscopy access during the pandemic has been reported elsewhere.^9^^,^^19^ On the other hand, some studies reported little impact of the COVID-19 pandemic on short-term stage shifts.^14^^,^^18^ CRC is generally recognized as slow-progressing cancer that takes a long time to double or progress,^20^ so it can be difficult to accurately capture the stage progression. In Japan, the 5-year survival rate for the localized stage is high, at more than 90%. Still, the survival rate decreases as the stage progress to about 60% for the regional stage and about 10% for the distant stage.^21^ Previous studies have shown that CRC survival is reduced by delays between diagnosis and treatment, and this has been shown to apply to any stage of the disease.^22^^–^^24^ The results also suggest that the risk of more advanced cancer is higher among females. This may be related to the higher rate of right-sided colon cancer in females, which is more likely to be detected at a more advanced stage, and also to differences in access to screening in females compared to males.^25^ Although the long-term effects of delayed treatment or stage progression during the pandemic are unknown, the results of various simulation studies predict that delayed diagnosis will increase excess mortality in the long term.^26^^–^^28^ It is crucial to predict future changes in these results and the ever-changing COVID-19 infection situation and to take countermeasures.

Our study has several limitations. First, the number of endoscopies and other tests needed for diagnosis cannot be ascertained from the data in this study. Second, longer-term observations are required to capture stage shifts in advanced cancers, and since the COVID-19 pandemic is still ongoing, such observation will be necessary. The impact of the pandemic on long-term prognosis also needs to be examined. Third, since the database used in this study is only a medical claims database and does not provide cancer-specific information, our findings may be a little less certain when compared to databases such as cancer registries. There may be problems of reduced diagnostic validity and misclassification for some of variables. The use of cancer registries is necessary to analyze changes in stage distribution in more detail. This study has limited generalizability, since the dataset only covers about 40% of DPC hospitals in Japan` and there may be differences from the stage distribution obtained from information in the National Cancer Registry, which covers cancer information for all of Japan.

In conclusion, we investigated the trends of stage distribution of CRC during the COVID-19 pandemic using the nationwide hospital-based claims database in Japan and found that the proportion of early-stage cancers tended to decrease temporarily after the initial spread of COVID-19 and the declaration of the state of emergency. While this effect may be temporary, the pandemic has since continued to waves of expansion and termination, stage distribution and long-term prognosis needs to be monitored closely in the future.

## SUPPLEMENTARY MATERIAL

eTable 1. The codes associated with specific surgery, endoscopic and radiotherapy treatment for CRC

eTable 2. Monthly stage trend before and during COVID-19 pandemic

eTable 3. Predicted percentage of monthly late stage before and during the COVID-19 pandemic
