# Supplementary material for: Trends in the Stage Distribution of Colorectal Cancer During the COVID-19 Pandemic in Japan: A Nationwide Hospital-claims Data Analysis
Source: J Epidemiol. 2024 Jul 5;34(7):356–61. doi: 10.2188/jea.JE20220347 (PMC11167262; doi:10.2188/jea.JE20220347)
Supplement: Supplementary file 1 [file je-34-356-s001.pdf]

eTable 1. The codes associated with specific surgery, endoscopic and radiotherapy treatment for CRC

Type of specific treatment for CRC	Procedure code
Surgical treatment with resection of primary lesion	
Open surgery	150064010, 150180950, 150181910, 150186810, 150186910, 150187210, 150187010, 150187110, 150190310, 150190710, 150245310, 150245410, 150264010, 150297510, 150420610
Laparoscopic surgery	150324910, 150325210, 150337810, 150337910
Robotic surgery	150407210, 150407310, 150407410
Endoscopic treatment	
Endoscopic mucosal resection	150183410, 150183650, 150285010, 150348510, 150348610, 150364510, 190181210, 190181310, 190181410, 190181510
Endoscopic submucosal dissection	150363910
Other surgical treatment	
	150180750, 150180850, 150181710, 150181810, 150183110, 150183510, 150184110, 150184310, 150184510, 150277810, 150322810, 150337710, 150361110, 150364010, 150377010, 150389610, 150402470, 150402570, 150420070,
Radiotherapy	
	180008810, 180019410, 180020710, 180020810, 180020910, 180021010, 180021110, 180021210, 180021310, 180021410, 180021510, 180021610, 180021710, 180021810, 180021910, 180022010, 180031910

CRC, colorectal cancer.

eTable 2. Monthly stage trend before and during COVID-19 pandemic

Month		Early stage		Late stage	
	Year	n	%	n	%
Jan					
	2018	860	(33.5)	1,708	(66.5)
	2019	879	(30.1)	2,037	(69.9)
	2020	1,018	(32.4)	2,128	(67.6)
Feb					
	2018	673	(30.0)	1,571	(70.0)
	2019	813	(29.2)	1,967	(70.8)
	2020	874	(30.2)	2,016	(69.8)
Mar					
	2018	635	(26.7)	1,741	(73.3)
	2019	832	(29.5)	1,993	(70.5)
	2020	951	(30.5)	2,172	(69.5)
Apr					
	2018	743	(28.9)	1,832	(71.1)
	2019	809	(30.0)	1,887	(70.0)
	2020	896	(29.9)	2,100	(70.1)
May					
	2018	723	(27.0)	1,958	(73.0)
	2019	784	(28.2)	1,995	(71.8)
	2020	754	(29.2)	1,828	(70.8)
Jun					
	2018	690	(25.3)	2,033	(74.7)
	2019	785	(27.1)	2,114	(72.9)
	2020	729	(25.9)	2,081	(74.1)
Jul					
	2018	730	(26.7)	2,008	(73.3)
	2019	873	(28.0)	2,249	(72.0)
	2020	617	(22.8)	2,094	(77.2)
Aug					
	2018	759	(27.0)	2,051	(73.0)
	2019	794	(27.6)	2,078	(72.4)
	2020	711	(25.8)	2,040	(74.2)
Sep					
	2018	682	(27.7)	1,782	(72.3)
	2019	846	(28.6)	2,116	(71.4)
	2020	693	(25.2)	2,056	(74.8)
Oct					
	2018	808	(28.0)	2,074	(72.0)
	2019	876	(29.2)	2,123	(70.8)
	2020	780	(26.7)	2,141	(73.3)
Nov					
	2018	778	(28.6)	1,939	(71.4)
	2019	877	(29.9)	2,061	(70.1)
	2020	866	(29.1)	2,108	(70.9)
Dec					
	2018	712	(28.5)	1,788	(71.5)
	2019	771	(28.1)	1,976	(71.9)
	2020	734	(29.1)	1,792	(70.9)

COVID-19, coronavirus disease 2019.

eTable 3. Predicted percentage of monthly late stage before and during the COVID-19 pandemic

	Before COVID-19		During COVID-19		
Month	Margin (%)	95% CI	Margin (%)	95% CI	Difference
January	68.1	[67.0, 69.0]	—	—	
February	70.4	[69.2, 71.7]	69.8	[68.1, 71.4]	-0.6
March	71.8	[70.6, 73.0]	69.5	[67.9, 71.2]	-2.3
April	70.6	[69.3, 71.8]	70.1	[68.5, 71.7]	-0.5
May	72.4	[71.2, 73.6]	70.8	[69.0, 72.6]	-1.6
June	73.8	[72.6, 74.9]	74.1	[72.4, 75.7]	0.3
July	72.6	[71.5, 73.8]	77.2	[75.7, 78.8]	4.6
August	72.7	[71.5, 73.8]	74.2	[72.5, 75.8]	1.5
September	71.8	[70.6, 73.0]	74.8	[73.2, 76.4]	3.0
October	71.4	[70.2, 72.5]	73.3	[71.7, 74.9]	1.9
November	70.7	[69.5, 71.9]	70.9	[69.2, 72.5]	0.2
December	71.7	[70.5, 73.0]	70.9	[69.1, 72.7]	-0.8

COVID-19, coronavirus disease 2019.

Multivariate logistic regression analysis adjusted for sex, body mass index, smoking index, and hospital scale.
